# Harnessing Digital Pathology Tools to Distinguish Hand Eczema From Palmar Psoriasis: A Quantitative Approach

**DOI:** 10.1111/cup.70064

**Published:** 2026-02-05

**Authors:** Melissa Sari, Stefan Schliep, Anne Petzold, Franklin Kiesewetter, Michael Erdmann, Michael Sticherling, Carola Berking, Markus V. Heppt, Elias A. T. Koch

**Affiliations:** ^1^ Department of Dermatology, Deutsches Zentrum Immuntherapie (DZI), Comprehensive Cancer Center (CCC) Erlangen‐EMN, Bavarian Cancer Research Center (BZKF) Uniklinikum Erlangen, Friedrich‐Alexander‐Universität Erlangen‐Nürnberg (FAU) Erlangen Germany; ^2^ MVZ Pathology Bamberg Germany; ^3^ DERMPATH München, Laboratory for Dermatopathology, Oral Pathology and Molecular Pathology Munich Germany

**Keywords:** dermatopathology, hand dermatitis, hand eczema, immunohistochemistry, palmar psoriasis, QuPath

## Abstract

**Background:**

Distinguishing hand eczema from palmar psoriasis is a common diagnostic challenge due to overlapping clinical and histopathological features.

**Objective:**

This study aimed to validate morphological and immunohistochemical criteria for differentiating these two conditions using the digital pathology tools *QuPath* and *ImageJ*.

**Methods:**

One hundred forty‐two histological samples with confirmed clinical diagnoses were stained with hematoxylin and eosin and subjected to immunohistochemical staining for CD3, CD15, CD20, CD123, S100, and PHH3. The samples were digitized for analysis. *QuPath* was used to automate annotation, segment epidermal layers, and measure rete ridge elongation, width, and suprapapillary epidermal thickness. Immunohistochemical markers were also analyzed using *QuPath*. *ImageJ* was employed to quantify spongiosis using color thresholding techniques.

**Results:**

Suprapapillary epidermal thickness was significantly lower in psoriasis compared to eczema (*p* < 0.001; AUC = 0.72). Rete ridge elongation (*p* = 0.002; AUC = 0.704) and width (*p* < 0.001; AUC = 0.698) also showed significant differences. Furthermore, hypogranulosis was more pronounced in psoriasis (*p* = 0.012; AUC = 0.602), while S100‐positive cells in the epidermis were more commonly observed in eczema (*p* = 0.013; AUC = 0.583).

**Conclusion:**

Quantitative assessment of suprapapillary epidermal thickness and rete ridge morphology offers a reliable and objective method for differentiating palmar psoriasis from hand eczema, enhancing diagnostic accuracy.

## 1 Introduction

Palmar skin conditions, particularly palmar psoriasis and hand eczema, are prevalent inflammatory dermatoses that profoundly affect patients' quality of life, resulting in functional limitations and social impairments [1]. Although hand eczema can arise from irritant, allergic, or atopic etiologies, and palmar psoriasis is driven by T‐cell‐mediated immune dysregulation and aberrant keratinocyte proliferation, these entities frequently exhibit overlapping clinical and histological features, making their distinction challenging [2, 3, 4, 5]. To make an accurate diagnosis, gathering detailed patient histories and performing a biopsy is often essential [6].

Histologically, psoriasis classically demonstrates confluent parakeratosis, thinning of the granular layer, spongiform pustules of Kogoj, psoriasiform hyperplasia, and the presence of neutrophil microabscesses of Munro [7]. Elongation of rete ridges and tortuous, dilated vessels in the papillary dermis are also typical, although such findings can be less distinct on the palms, particularly when superimposed irritant or allergic factors alter the usual psoriatic morphology [8]. Meanwhile, chronic hand eczema commonly displays spongiosis, microvesicles, edema within the papillary dermis, and plasma mounds [9]. However, longstanding lesions may acquire psoriasiform features, including irregular rete ridge hyperplasia or focal loss of the granular layer [10, 11]. Inter‐observer variability in the interpretation of the histopathological criteria remains a challenge, emphasizing the need for more objective and reproducible diagnostic methods [12].

In recent years, machine learning technologies and digital image analysis have gained attention in medical diagnostics, particularly in histopathology [13]. One such tool, *QuPath*, has been developed as an open‐source platform for digital pathology, enabling automated annotation, segmentation, and quantification of tissue features in scanned histological slides [14]. *ImageJ*, another widely adopted open‐source environment, enables a broad spectrum of biomedical image processing techniques [15].

In this study, we evaluated defined histological features and immunohistochemically stained slides to assess their diagnostic utility in a computer‐assisted standardized manner.

## 2 Materials and Methods

### 2.1 Study Design and Patient Selection

**FIGURE 1:**
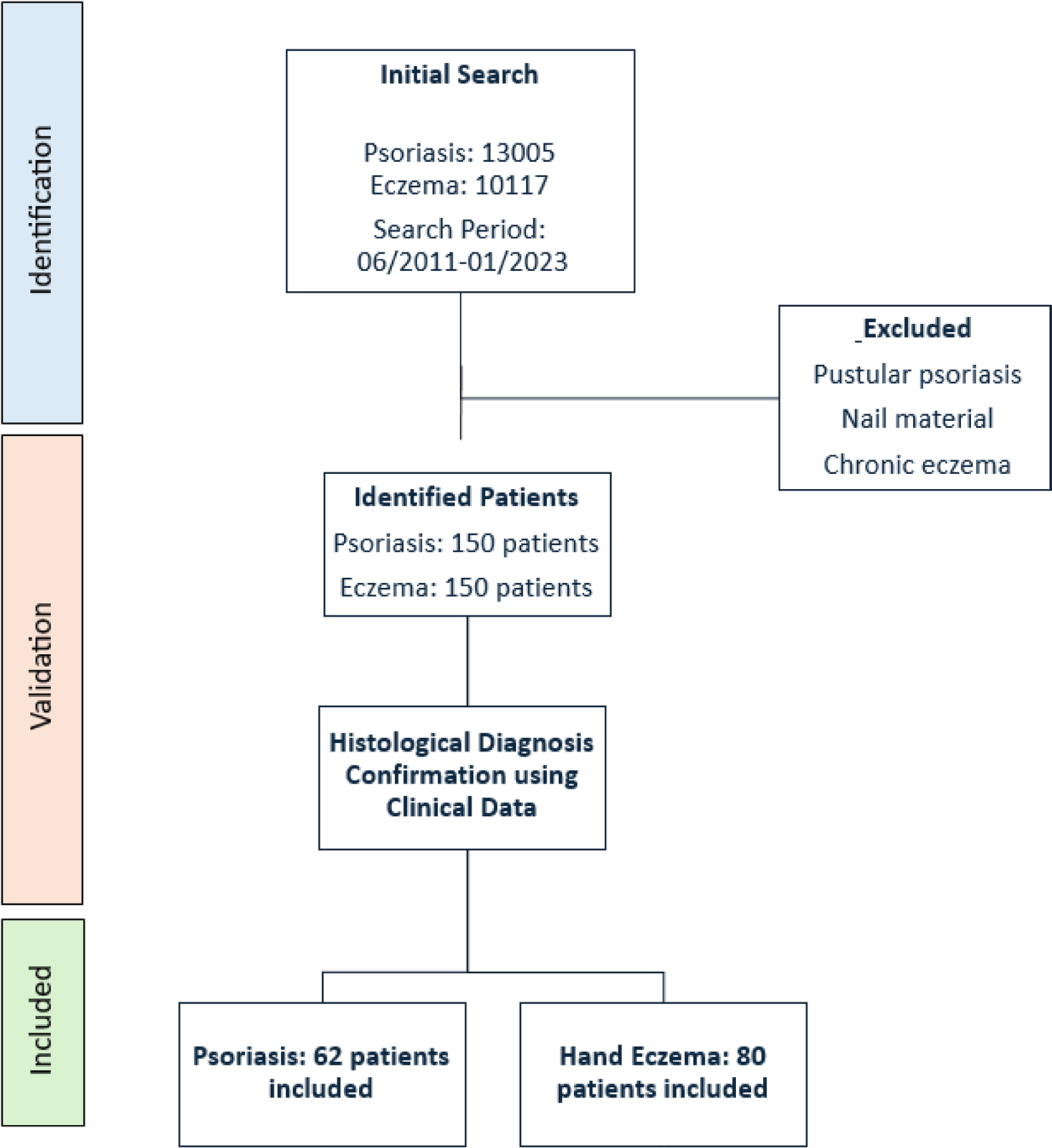
Flowchart of the patient selection process in the study. An initial search in the internal biopsy database identified 13 005 psoriasis and 10 017 eczema cases (2011–2023). Clinical verification ensured that histopathological diagnoses aligned with clinical data from electronic patient records. Subsequently, *n* = 62 psoriasis and *n* = 80 eczema cases were confirmed for analysis.

A systematic search was conducted in our clinical database of previously performed skin biopsies. For both groups, inclusion criteria encompassed biopsies taken from the “hand,” “finger,” or “palmar” regions. In total, 13 005 psoriasis and 10 117 eczema cases were screened to identify a preliminary cohort of 150 patients with psoriasis of the hand and 150 patients with hand eczema. Subsequently, the initial histological diagnoses were cross‐referenced with clinical data stored in the patient record charts. After validation with clinical data, 62 psoriasis cases and 80 eczema cases were confirmed and included in the analysis (Figure 1). Ambiguous cases or cases with discrepancies between histopathology and the clinical presentation were excluded.

### 2.2 Immunohistochemistry and Hematoxylin and Eosin (H&E) Staining

Immunohistochemistry and H&E staining were performed in the certified laboratory of the Unit for Dermatohistology, Uniklinikum Erlangen, Germany, using a fully automated slide staining instrument (BenchMark XT by Roche Diagnostics, Rotkreuz, Switzerland) and Histocore‐Spectra by Leica Biosystems. The markers for IHC included CD3 for T lymphocytes, CD15 for neutrophils, CD20 for B lymphocytes, CD123 for plasmacytoid dendritic cells, S100 for Langerhans cells and melanocytes, and pHH3 for mitotic activity indicating cell proliferation. Primary antibodies were applied according to the manufacturers' recommended dilutions and incubation times. Visualization of antibody binding was achieved using Ultra View Red 250 Tests (760–501) by Roche Diagnostics as chromogen. The CD3 antibody (Cell Marque MRQ‐39, rabbit monoclonal) was diluted 1:200 and incubated for 36 min. Confirm anti‐CD15 (MMA) ready‐to‐use mouse monoclonal antibody by Ventana Roche was incubated for 16 min. The CD20 antibody (Clone L26 Anti‐Human CD20cy by Dako, mouse monoclonal) was diluted 1:500 and incubated for 44 min. S100, a ready‐to‐use polyclonal antibody, was incubated for 32 min. Phosphohistone H3 (pHH3, rabbit polyclonal, Cell Marque) was diluted 1:200 and incubated for 36 min.

### 2.3 Image Data Acquisition and Analysis

The slides were scanned at 40× magnification with Precipoint, FRITZ producing images at a resolution of 0.2156 μm per pixel. For analysis of the slides, the bioimage software QuPath (version 0.4.1) was used.

### 2.4 Automated Annotation and Segmentation of Epidermal Layers

**FIGURE 2:**
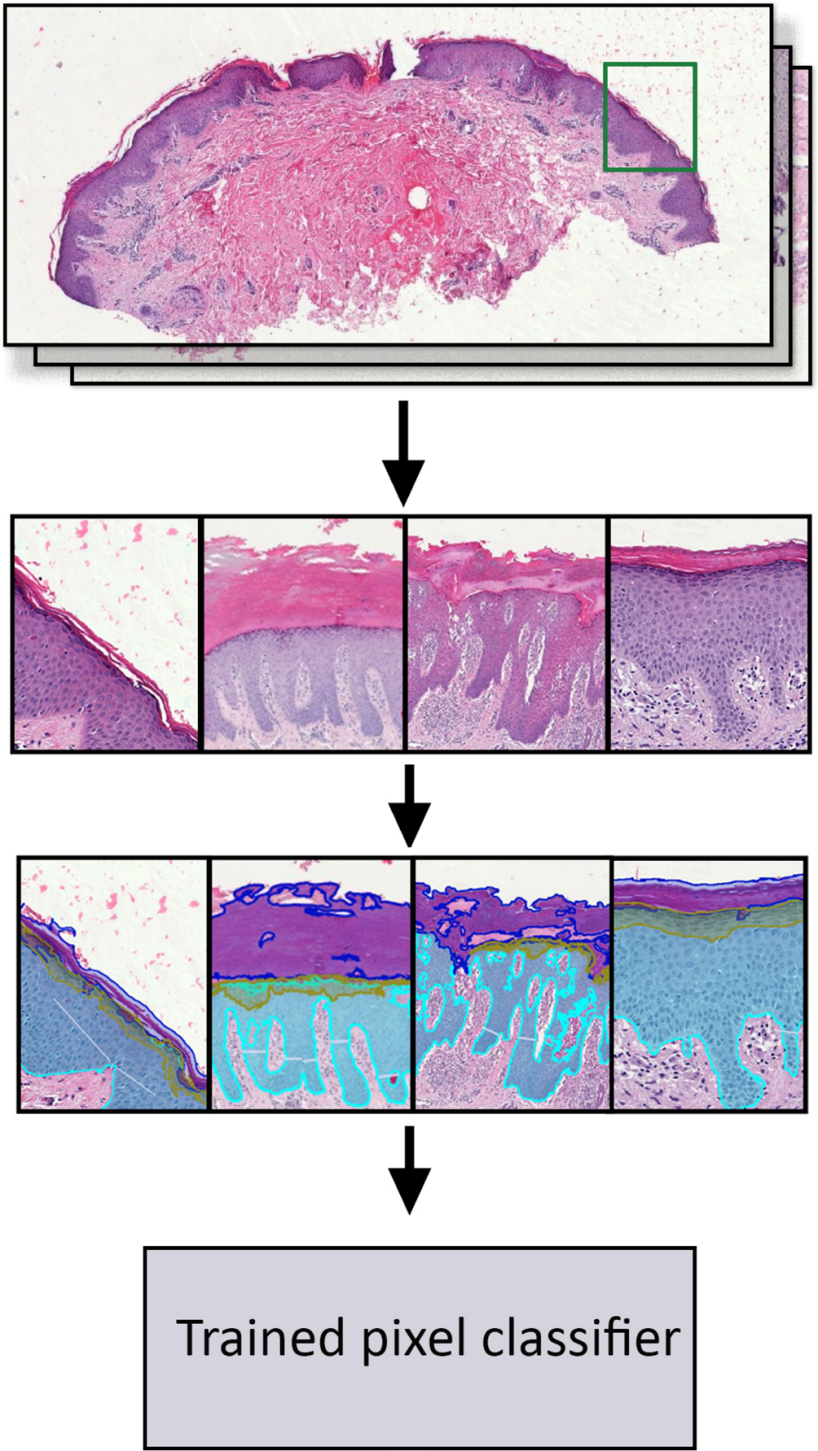
Automated pixel classification process using *QuPath* software for epidermal layer segmentation. In the first step, representative regions were selected from H&E‐stained histological sections to construct a composite training image. In the second step, these regions within the training image were manually annotated to delineate the specific layers. These manually created annotations, shown in the middle panel, served as the training dataset for the *QuPath* pixel classifier. The bottom panel illustrates the application of the trained classifier to create annotations for epidermal layer segmentation on all H&E slides.

For automated annotation, a representative histological section was manually labeled to delineate epidermal layers: corneal layer, granular layer, and the remaining epidermis. These annotations served as the training dataset for *QuPath* and were used to calculate the ratios of stratum granulosum, stratum corneum, and spongiosis relative to the total epidermis (Figure 2).

### 2.5 Quantitative Measurement of Histopathological Features

The choice of histopathological parameters for quantitative analysis was guided by well‐defined criteria in dermatopathology literature. Suprapapillary epidermal thinning, elongation of rete ridges, and hypogranulosis are consistently described as key features of psoriasis [7, 8, 16, 17]. Rete ridge elongation is particularly emphasized in psoriasis [17]. Capillary diameter, spongiosis, and parakeratosis were selected due to frequent mention in previous studies [7, 8, 16, 17].

#### 2.5.1 Evaluation of Rete Ridge Morphology

Rete ridge elongation was measured as the vertical distance from the apex of the dermal papilla to the deepest point of the rete ridge at the epidermal‐dermal junction. Three representative ridges per slide were measured to ensure consistency. The width of the rete ridges was assessed by measuring the horizontal distance between their lateral edges at the basal boundaries within the epidermal‐dermal junction. These boundaries were defined by the points where the rete ridges are adjacent to neighboring dermal papillae. Up to 15 measurements were performed per slide to account for morphological variability, with a minimum of five measurements required per slide to ensure reliability.

#### 2.5.2 Capillary Assessment

The assessment of the capillaries within dermal papillae was determined by measuring the maximum visible longitudinal length of capillaries in the histological plane, with straighter, less coiled capillaries appearing longer. Measurements were performed on three representative papillae per slide.

#### 2.5.3 Parakeratosis

Parakeratosis was assessed by measuring the start and end points of parakeratotic areas within the stratum corneum. For multiple foci, each was measured individually and their average was used to represent the overall extent per sample.

#### 2.5.4 Suprapapillary Epidermal Thickness

The suprapapillary epidermal thickness was measured as the vertical distance from the apex of the dermal papilla to the lowest point of the epidermis beneath the stratum corneum, reflecting the viable epidermis. Measurements were averaged from three to five representative areas per slide.

#### 2.5.5 Spongiosis

**FIGURE 3:**
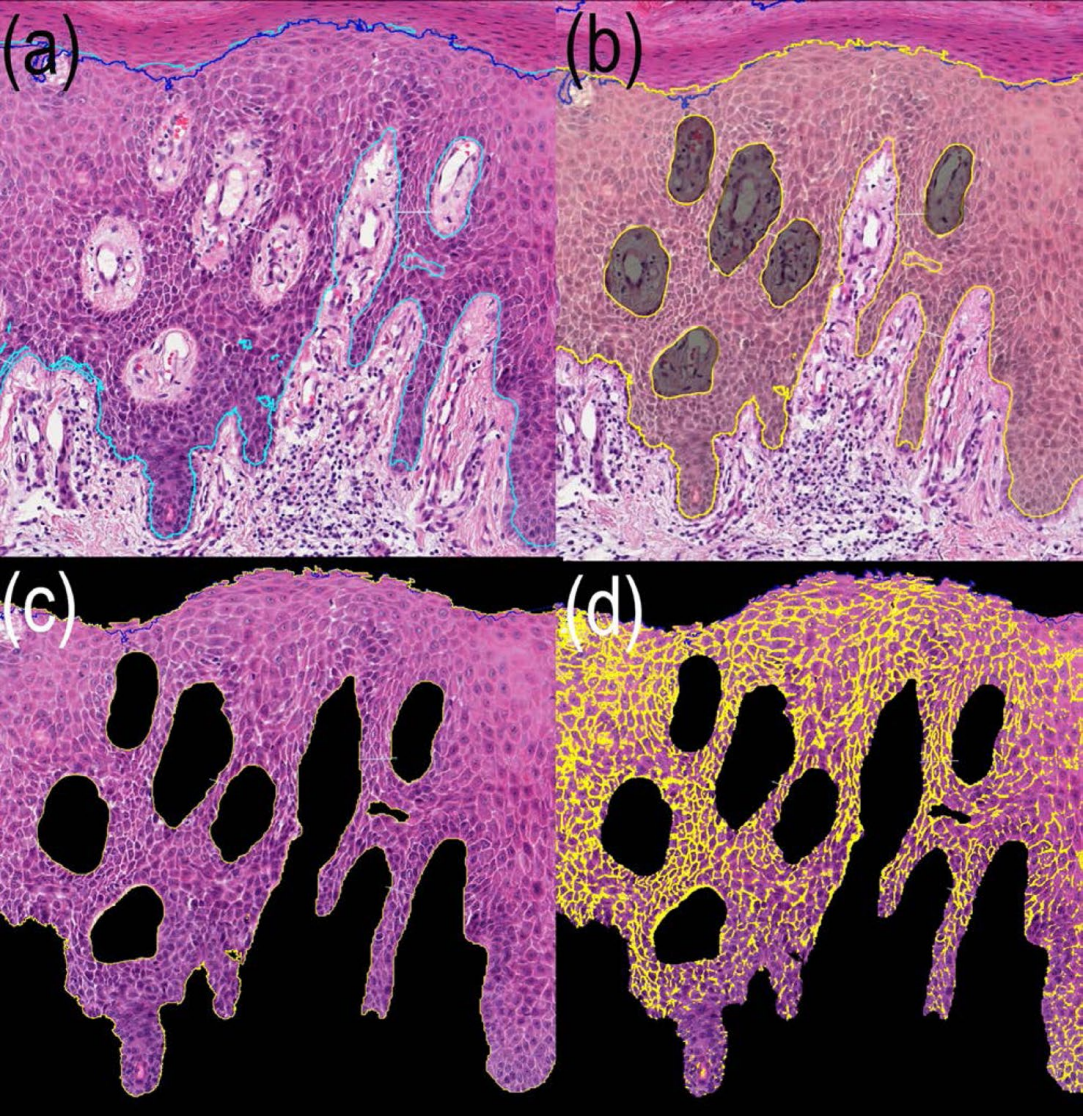
Histological section of psoriatic skin (H&E stain, 40×) showing different stages of digital segmentation and analysis for the quantification of spongiosis using QuPath and ImageJ. (a) Shows the initial annotation of the epidermis, excluding the stratum corneum, performed using *QuPath* software. (b) Illustrates the exported annotated image with additional exclusion of subepidermal artifacts (darkened areas). (c) Depicts the region of interest defined for analysis in *ImageJ*, where the color threshold function is subsequently applied to quantify intercellular edema. (d) Shows the final quantified spongiotic regions, with the highlighted areas overlaid on the epidermis.

For spongiosis quantification, the epidermis (excluding the stratum corneum) was annotated in *QuPath* and exported to *ImageJ*. Using the “color threshold” function, intercellular oedema was identified as bright spongiotic areas, while white regions were excluded to avoid artifacts. This ensured precise detection of relevant spaces. Spongiosis extent was calculated as the area of spongiotic spaces relative to the total annotated epidermis (Figure 3).

### 2.6 Immunohistochemistry

**FIGURE 4:**
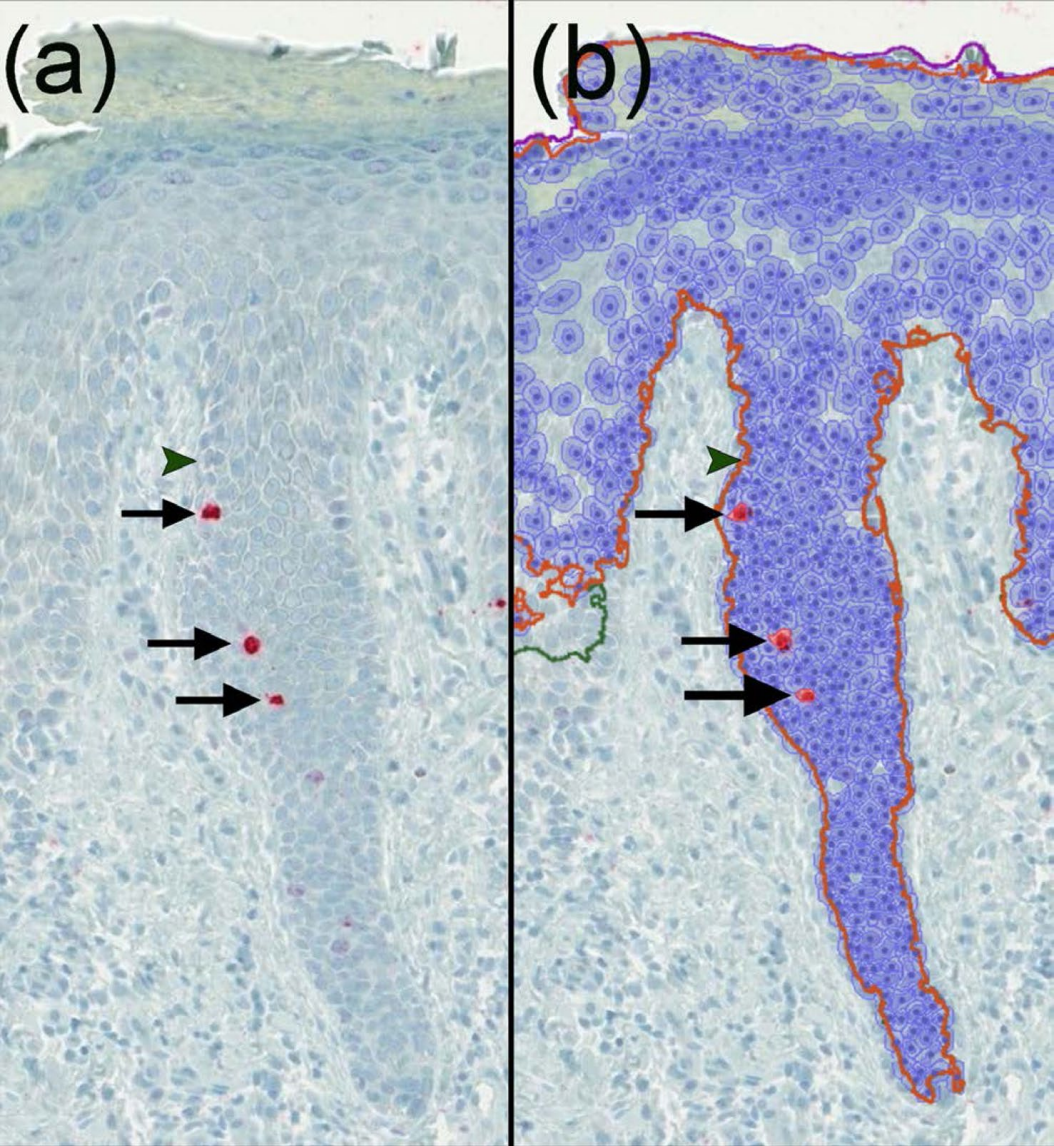
Representative image of pHH3 immunofluorescence staining (40×) in hand eczema. (a) Shows the area without annotation. (b) Illustrates the detection of pHH3‐positive cells (marked with arrows) within the automatically annotated epidermis. An exemplary pHH3‐negative cell is marked with an arrowhead.

Quantitative assessment of immunoreactivity was performed using *QuPath* software. Positive cells were identified and counted in representative areas of the epidermis and dermis. The density of positive cells was calculated as the number of positive cells per square millimeter (cells/mm^2^) (Figure 4).

### 2.7 Statistical Analysis

Statistical analyses assessed the diagnostic utility of key histopathological parameters. Diagnostic groups were compared using independent *t*‐tests, with *p* < 0.05 considered significant. ROC curve analysis evaluated the accuracy of each parameter, with AUC calculated accordingly. Multivariate analysis via general linear models assessed the combined effect of multiple variables, applying Pillai's Trace, Wilks' Lambda, Hotelling's Trace, and Roy's Largest Root for significance testing. All analyses were conducted using IBM SPSS Statistics (v28.0.0.0).

## 3 Results

A total of 142 histological specimens were analyzed, comprising 80 cases of palmar psoriasis and 62 cases of hand eczema. The baseline characteristics are summarized in Table 1. Due to technical artifacts, sectioning issues, or poor sample quality, several parameters were not assessable in all 142 cases. Missing values are listed in Table 2.

**TABLE 1:** Baseline characteristics of the study cohort.

Characteristics		Eczema (*n* = 80)	Psoriasis (*n* = 62)	*p*
Age, mean, years (SD)		52.8 (16.3)	57.7 (13.3)	0.051
Female, *n* (%)		39 (27.5%)	23 (16.2%)	0.223
Location, *n* (%)	Digits	14 (9.9%)	8 (5.6%)	0.492
	Wrist	13 (9.1%)	4 (2.8%)	0.116
	Palm	43 (30.3%)	24 (16.9%)	0.091
	Dorsal hand	10 (7.0%)	20 (14.0%)	0.007
	Not further specified	0 (0.0%)	6 (4.2%)	0.006

**TABLE 2:** Results.

Parameter	Group	*N*	Missing values (*N*)	Mean	Std. error mean	ROC/AUC	*p* (*t*‐test)
Elongation of the rete ridges (μm)	Eczema	37	43	309.64	23.56	0.704	0.002
	Psoriasis	35	27	410.21	25.48		
Suprapapillary epidermal thickness (μm)	Eczema	74	6	78.93	3.31	0.720	< 0.001
	Psoriasis	62	0	59.82	4.53		
Width of the parakeratosis (μm)	Eczema	30	50	1652.09	240.37	0.544	0.183
	Psoriasis	34	28	2040.94	365.70		
Capillary diameter (μm)	Eczema	26	54	108.30	8.76	0.721	0.067
	Psoriasis	19	43	272.87	104.49		
Width of the rete ridges (μm)	Eczema	73	7	81.84	3.28	0.698	< 0.001
	Psoriasis	62	0	63.76	3.00		
Ratio stratum granulosum to epidermis (μm^2^/μm^2^)	Eczema	76	4	0.13	0.01	0.602	0.012
	Psoriasis	62	0	0.10	0.06		
Ratio stratum corneum to epidermis (μm^2^/μm^2^)	Eczema	76	4	0.69	0.08	0.508	0.442
	Psoriasis	62	0	0.71	0.13		
Ratio of spongiosis to epidermis (μm^2^/μm^2^)	Eczema	77	3	0.21	0.01	0.604	0.345
	Psoriasis	62	0	0.2	0.02		
CD3 (cells/μm^2^)	Eczema	69	11	65.34	10.32	0.533	0.323
	Psoriasis	61	1	59.38	7.32		
CD15 (cells/μm^2^)	Eczema	58	22	42.64	18.86	0.674	0.245
	Psoriasis	59	3	58.89	13.77		
CD20 (cells/μm^2^)	Eczema	72	8	3.12	0.90	0.605	0.061
	Psoriasis	57	5	6.44	1.93		
PHH3 (cells/μm^2^)	Eczema	71	9	23.14	4.01	0.604	0.107
	Psoriasis	62	0	30.03	3.72		
S100 (cells/μm^2^)	Eczema	65	15	101.84	14.33	0.583	0.013
	Psoriasis	58	4	64.04	8.78		

### 3.1 Morphologic Features

Among the evaluated parameters, suprapapillary epidermal thickness was 59.82 μm (±4.53 standard error mean = SEM) in psoriasis compared to 78.93 μm (±3.31 SEM) in eczema (*p* < 0.001, AUC = 0.720). Elongation of the rete ridges measured 410.21 μm (±25.48 SEM) in psoriasis versus 309.64 μm (±23.56 SEM) in eczema (*p* = 0.002, AUC = 0.704). Further, rete ridge width (*p* < 0.001, AUC = 0.698) and the ratio of stratum granulosum to epidermis (*p* = 0.012, AUC = 0.602) were significantly different in both conditions. Capillary extension (*p* = 0.067, AUC = 0.721) demonstrated a statistical trend. For a full picture, see Table 2 and Figure S1.

### 3.2 Immunohistochemical Analysis

Among the immunohistochemical markers, S100 was the only one to show a statistically significant difference, with values of 101.84 cells/mm^2^ (±14.33 SEM) in eczema versus 64.04 cells/mm^2^ (±8.78 SEM) in psoriasis (*p* = 0.013, AUC = 0.583). For a comprehensive overview, see Table 2 and Figure S2.

### 3.3 Subgroup Analysis

Subgroup analysis was performed to investigate whether suprapapillary thickness and rete ridge morphology remained consistent after exclusion of cases with secondary morphologic alterations. After excluding cases with eczematized features—defined as superimposed lymphocytic infiltration, serum crusts, or other secondary histological changes—as well as samples with impetiginized features and psoriasis with pustules, 128 cases remained (*n* = 73 hand eczema, *n* = 55 palmar psoriasis). Purely pustular psoriasis was generally excluded from the analysis. Overall findings were similar to the main cohort. Notably, pHH3 achieved statistical significance in this refined subgroup, while it was not significant in the main analysis (*p* = 0.011, AUC = 0.626) (Tables S1 and S2).

### 3.4 Multivariate Analysis

A comprehensive multivariate analysis was conducted on the entire dataset of 136 (*n* = 73 hand eczema, *n* = 62 psoriasis) to assess whether suprapapillary epidermal thickness and rete ridge width were independent, distinctive features. The observed power in the corrected model was 0.95 for suprapapillary epidermal thickness and 0.979 for the width of the rete ridges (Table 3 and Figure S3).

**TABLE 3:** Multivariate analysis.

Parameter	Eczema *n* = 73 mean (SD) μm	Psoriasis *n* = 63 mean (SD) μm	*F* (corrected model)	Observed power (corrected model)	Observed power (group)
Suprapapillary epidermal thickness	79.72 (27.84)	59.82 (35.92)	13.208	0.950	0.950
Width of the rete ridges	81.84 (28.05)	63.76 (23.84)	16.122	0.979	0.979

## 4 Discussion

Inflammatory conditions affecting the palms continue to pose a diagnostic challenge, particularly in differentiating psoriasis from hand eczema. Extrinsic factors such as mechanical stress and exposure to irritants or allergens can blur the clinical and histological presentation [16]. Psoriatic lesions on the palms may appear more hyperkeratotic and less clearly demarcated, while chronic hand eczemas can develop psoriasiform hyperplasia or parakeratotic changes after repeated irritation or partial treatments [17]. Conversely, classical findings of hand eczema, such as spongiosis or plasma mounds, may also appear in certain psoriatic conditions [18]. Consequently, this overlap has prompted multiple studies investigating refined histopathological and immunohistochemical approaches and criteria [9].

In our cohort, suprapapillary epidermal thinning and elongated, narrow rete ridges emerged as the most powerful and consistently discriminative features for psoriasis, remaining robust even after excluding cases with secondary alterations in the subgroup analysis. A lower stratum granulosum‐to‐epidermis ratio further highlighted hypogranulosis as a diagnostic clue. Together, these parameters represent the strongest histopathologic features of psoriasis and are readily appreciable in routine practice, giving them direct diagnostic value for dermatopathologists. In contrast, spongiosis and parakeratosis showed broad overlap between the entities with only minimal additional diagnostic value. Among immunomarkers, epidermal S100‐positive cells were more common in eczema. pHH3 reached significance only in analyses restricted to cases without irritation or infection and provided less diagnostic value than the morphometric markers.

In a study of 17 clinically confirmed palmoplantar psoriasis and 25 eczematous dermatitis cases, Aydin et al. identified a significant difference only with vertically oriented parakeratotic foci alternating with orthokeratosis, which were observed in 76.5% of psoriasis versus 32% of eczema cases (*p* = 0.005) [18]. Other parameters, including spongiosis, acanthosis, and irregular rete ridge patterns, were not significantly different [18]. Similarly, Kamyab Hesari et al. analyzed 36 palmoplantar psoriasis and 16 eczema cases, identifying six markers favoring psoriasis: hypogranulosis, Munro's microabscesses, tortuous dermal vessels (each *p* < 0.001), suprapapillary thinning (*p* = 0.02), confluent parakeratosis (*p* = 0.044), and spongiform pustules (*p* = 0.047). In contrast, plasma mounds were more common in eczema (*p* = 0.022) [16]. Their identification of suprapapillary thinning and hypogranulosis as markers of psoriasis closely aligns with our findings. Similarly, the conclusion of Aydin et al. that spongiosis was not discriminative is consistent with our results. In both studies, diagnoses were clinically confirmed and followed by manual H&E evaluation by experienced dermatopathologists [18].

Further, Park et al. conducted a retrospective review of 96 cases, comprising palmar psoriasis (*n* = 30), hand eczema (with or without atopic/nummular features; *n* = 31), and hyperkeratotic hand dermatitis (*n* = 35) [17]. They used a systematic morphological approach that included assessment of the granular layer (e.g., reduced or absent), psoriasiform epidermal hyperplasia, and the presence or degree of parakeratosis or spongiosis. Their findings highlighted a reduced or absent granular layer as being significantly more frequent in palmar psoriasis than in the other subgroups (*p* = 0.047 for psoriasis vs. hand eczema; *p* = 0.007 for psoriasis vs. hyperkeratotic dermatitis). These findings support previous observations that a thinned granular layer often correlates with psoriatic pathology [17]. Consistent with these observations, our study also demonstrated pronounced hypogranulosis in psoriatic lesions. In addition, Park et al. observed spongiosis and parakeratosis across all three groups, underscoring the overlap, which is in line with our results [17].

Notably, An et al. offered a broader, more immunological perspective by evaluating 66 patients with palmar psoriasis, chronic hand eczema (CHE), and hyperkeratotic hand dermatitis (HHD) [19]. Confluent parakeratosis occurred significantly more often in palmar psoriasis than in CHE (*p* < 0.001) or HHD (*p* = 0.028). Loss of the granular layer and psoriasiform epidermal hyperplasia were both significantly more frequent in palmar psoriasis and HHD than in CHE (*p* < 0.001). Although spongiosis is typically tied to eczematous conditions, An et al. observed it in 61.9% of palmar psoriasis, 35.0% of CHE, and 30.0% of HHD cases (*p* = 0.033). On the immunohistochemical level, elevated expression of HBD2 and IL‐36γ in HHD and psoriasis suggested shared immunopathological pathways, distinguishing these groups from CHE [19].

The differences between our results and those of Park et al. and An et al. may be partly explained by the differing methods and the heterogeneous composition of Park's cohort. Although Park and colleagues systematically assessed histopathological features, they did so largely using a descriptive or semi‐quantitative approach (e.g., grading spongiosis as mild, moderate, or severe). This heterogeneity, while more reflective of real‐world variability, may have diluted specific distinctions that emerge when focusing on a narrower, well‐validated subset.

Cesinaro et al. combined histological and immunohistochemical analysis to differentiate psoriasis from allergic contact dermatitis (ACD) in a cohort of 42 patients [20]. They evaluated regular vs. irregular epidermal hyperplasia (reflecting uniform vs. variable rete ridge elongation), the extent of parakeratosis (from focal to confluent), keratinocytic proliferation via Ki‐67/Mib‐1, and the density of S100‐positive dendritic cells. Regular hyperplasia and marked parakeratosis were more frequent in psoriasis, while ACD showed irregular hyperplasia and significantly higher S100‐positive dendritic cell counts (*p* = 0.006). Their observation of uniform rete ridge elongation in psoriasis aligns with our findings. Similarly, the higher S100 expression in ACD reflects our observation of increased S100 in eczema.

Complementary to these histopathological criteria, recent studies established a molecular classifier to distinguish psoriasis from eczema based on RT‐PCR analysis of NOS2 and CCL27 expression, achieving diagnostic accuracies above 95% [21]. Nevertheless, a morphology‐based diagnosis remains the primary approach in the vast majority of cases as it is easier, faster, and less cost intensive than the molecular classifier, highlighting the continued relevance of the assessment of morphologic features in routine clinical practice [21, 22, 23].

Recent publications along the IL‐17/IL‐36 axis support the utility of IL‐36 as a diagnostic marker for psoriasis [24]. Notably, one study focusing on the differential diagnosis of palmoplantar eczema and palmoplantar psoriasis reported stronger IL‐36 expression in the upper epidermis of psoriasis. However, that work was limited by a semiquantitative assessment [25]. Although we did not evaluate IL‐36 in our cohort, the sampling and *QuPath*‐based workflow we describe here would readily accommodate IL‐36 in future studies, enabling quantitative, multi‐observer analyses to further investigate this marker's incremental diagnostic value.

Despite integrating digital pathology and clinical cross‐verification, our retrospective, single‐center design introduces potential selection bias and limits broader generalizability. Immunostaining results may vary with disease stage, prior treatments, or concurrent infections, which can reduce the reliability of single markers. Some parameters were unassessable due to artifacts or incomplete sections. Furthermore, our measurement of “capillary extension” does not represent a strict perpendicular vessel diameter. Instead, we assessed the maximum visible longitudinal extension of the capillaries within the histological section. Thus, although indirect, this approach captures differences in vascular morphology.

Nonetheless, a major strength of our study lies in its standardized, quantitative histopathological evaluation, which minimizes subjectivity and enables objective detection of subtle epidermal differences. This was reinforced by thorough clinical validation. Compared to earlier approaches, our method allowed for more consistent identification of morphological features. A key advantage was *QuPath* automated annotation and quantification of defined epidermal layers, reducing observer variability and enhancing reproducibility across cases [26].

## 5 Conclusion

Building on previous work, we translate classic histopathologic features into quantitative measurements using open‐source tools (*QuPath/ImageJ*) in a clinically verified cohort, identifying suprapapillary epidermal thickness and rete ridge morphology as consistent, quantifiable parameters for distinguishing palmar psoriasis from hand eczema. Hypogranulosis was also more frequent in psoriasis but showed lower diagnostic power than suprapapillary thickness or rete ridge morphology. Although immunohistochemistry added limited value, digital pathology with automated annotation significantly improved reproducibility and may guide future diagnostic strategies. Future studies should define precise quantitative ranges for these parameters to facilitate reliable individual sample evaluation.

## Author Contributions

**M.S.:** data curation, formal analysis, writing first draft. **E.A.T.K.:** conceptualization, methodology, writing – review and editing. **M.V.H.:** supervision, writing – review and editing. All other authors writing – review and editing.

## Funding

The authors have nothing to report.

## Ethics Statement

This study was approved by an independent research ethics committee of the Friedrich‐Alexander‐Universität Erlangen‐Nürnberg (FAU; approval number 22‐368‐Br) and was conducted following the principles of the Helsinki Declaration in its current version.

## Conflicts of Interest

The authors declare no conflicts of interest.

## Supporting information


**Figure S1:** Receiver Operating Characteristic (ROC) curves for key histopathological parameters. (a) Capillary diameter (AUC 0.721); (b) suprapapillary epidermal thickness (AUC 0.72); (c) elongation of the rete ridges (AUC 0.704); (d) width of the rete ridges (AUC 0.698); (e) ratio stratum granulosum to epidermis (AUC 0.602); (f) ratio of spongiosis to epidermis (AUC 0.552); (g) width of the parakeratosis (AUC 0.544); (h) ratio stratum corneum to epidermis (AUC 0.508).

**Figure S2:** Receiver Operating Characteristic (ROC) curves for immunohistochemical markers. (a) CD15 (AUC 0.674); (b) CD20 (AUC 0.605); (c) PHH3 (AUC 0.604); (d) S100 (AUC 0.583); (e) CD3 (AUC 0.533).

**Figure S3:** Bar plots showing the estimated marginal means (in μm) from the multivariate analysis. Suprapapillary epidermal thickness for hand eczema (green) and palmar psoriasis (blue). Width of the rete ridges for hand eczema (green) and palmar psoriasis (blue).

**Table S1:** Subgroup analysis.
**Table S2:** Results subgroup analysis.

## Data Availability

The data that support the findings of this study are available on request from the corresponding author. The data are not publicly available due to privacy or ethical restrictions.
