# Supplementary figures and images for: Harnessing Digital Pathology Tools to Distinguish Hand Eczema From Palmar Psoriasis: A Quantitative Approach
Source: J Cutan Pathol. 2026 Feb 5;53(5):444–53. doi: 10.1111/cup.70064 (PMC13040410; doi:10.1111/cup.70064)

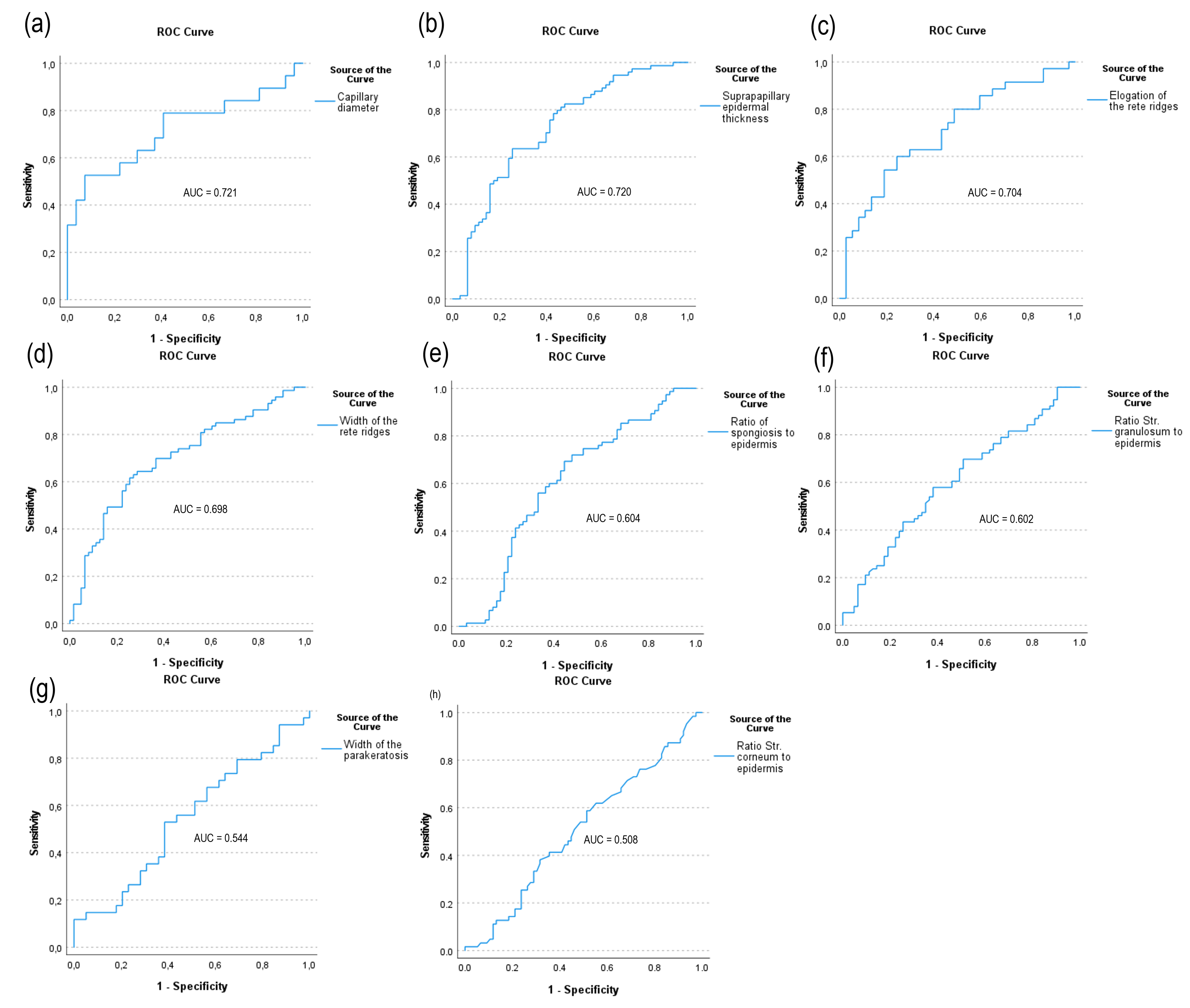

Supplement: Supplementary file 1 — Figure S1: Receiver Operating Characteristic (ROC) curves for key histopathological parameters. (a) Capillary diameter (AUC 0.721); (b) suprapapillary epidermal thickness (AUC 0.72); (c) elongation of the rete ridges (AUC 0.704); (d) width of the rete ridges (AUC 0.698); (e) ratio stratum granulosum to epidermis (AUC 0.602); (f) ratio of spongiosis to epidermis (AUC 0.552); (g) width of the parakeratosis (AUC 0.544); (h) ratio stratum corneum to epidermis (AUC 0.508). [file CUP-53-444-s004.jpg]

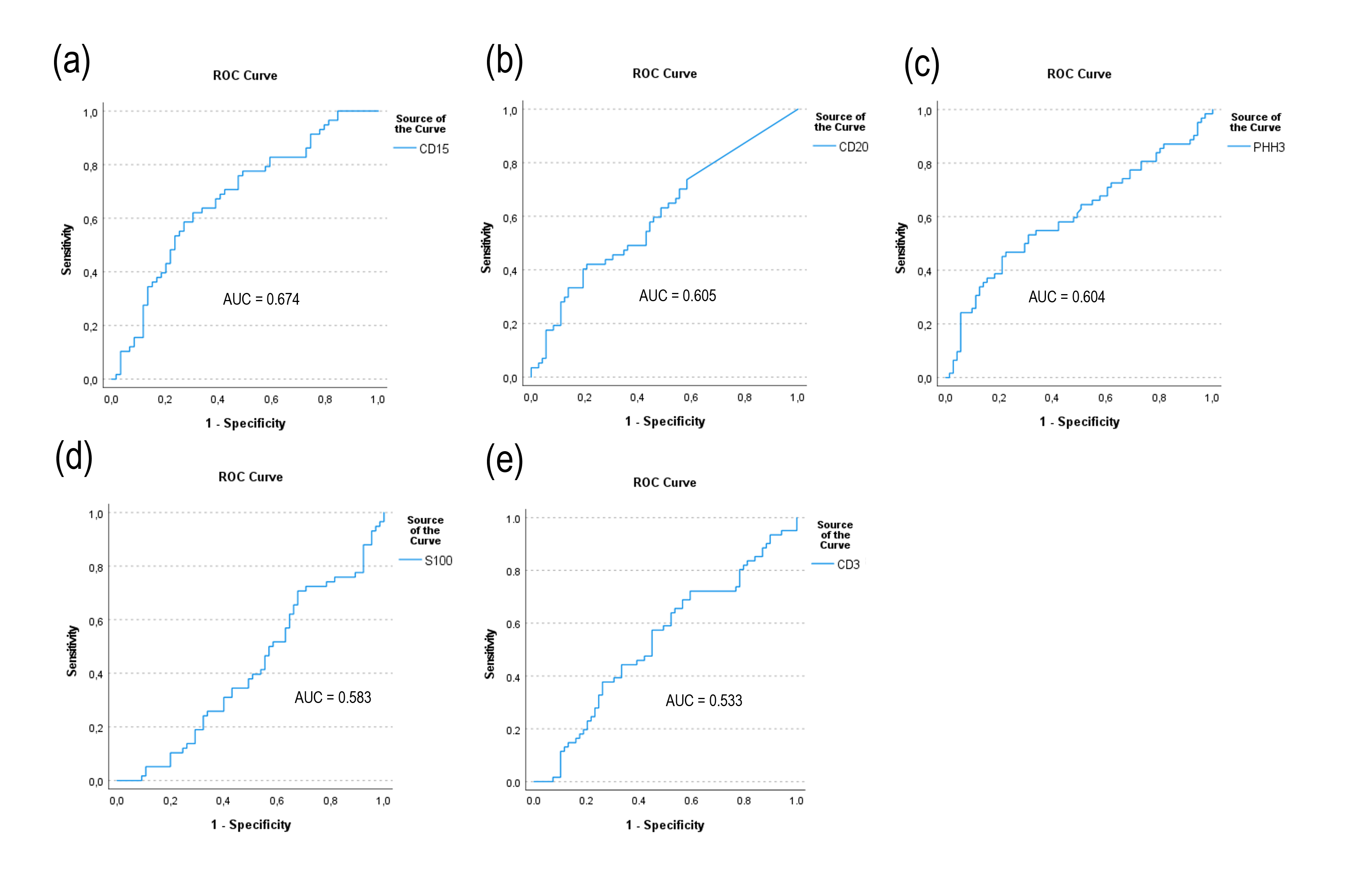

Supplement: Supplementary file 2 — Figure S2: Receiver Operating Characteristic (ROC) curves for immunohistochemical markers. (a) CD15 (AUC 0.674); (b) CD20 (AUC 0.605); (c) PHH3 (AUC 0.604); (d) S100 (AUC 0.583); (e) CD3 (AUC 0.533). [file CUP-53-444-s001.jpg]

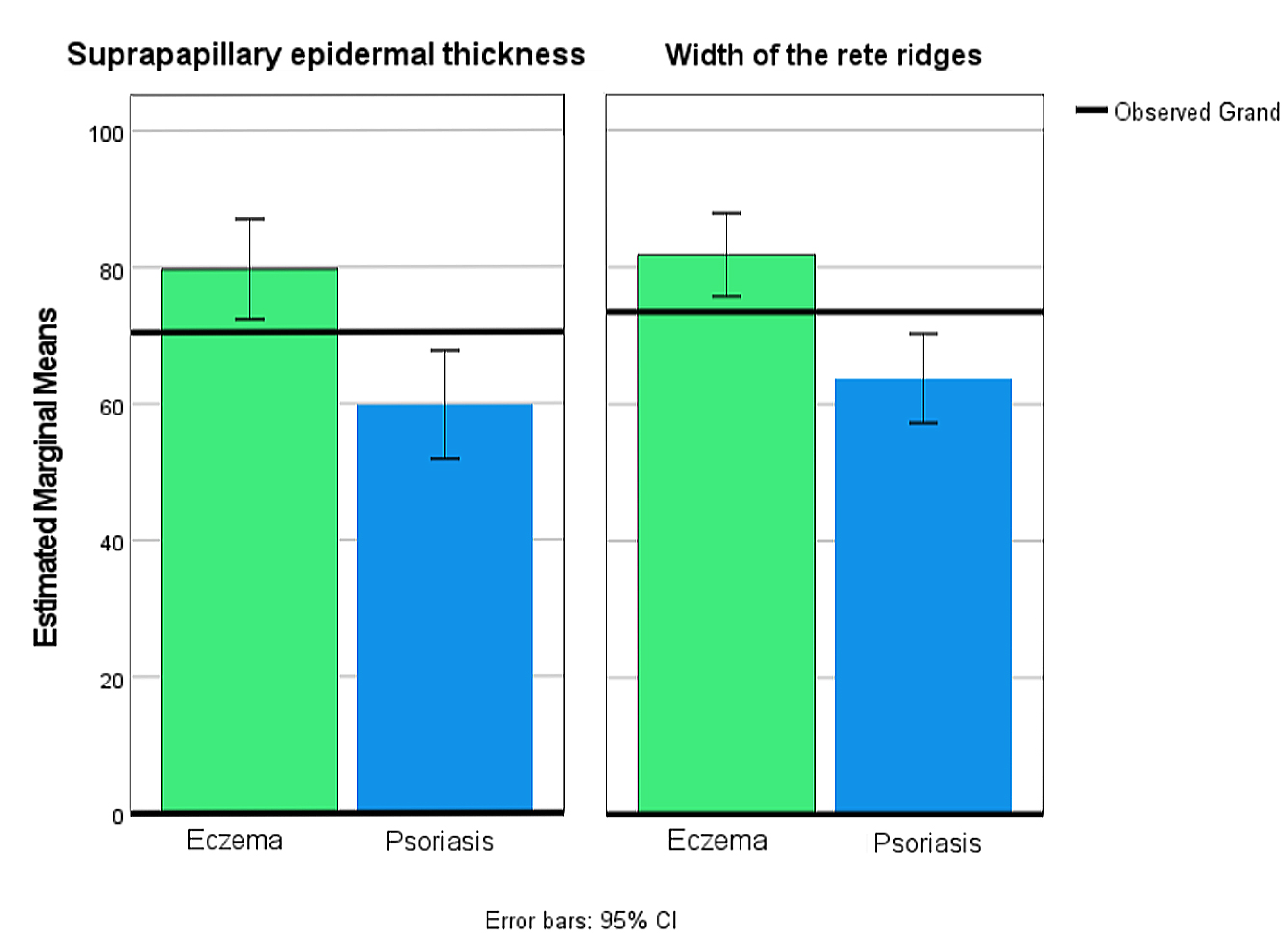

Supplement: Supplementary file 3 — Figure S3: Bar plots showing the estimated marginal means (in μm) from the multivariate analysis. Suprapapillary epidermal thickness for hand eczema (green) and palmar psoriasis (blue). Width of the rete ridges for hand eczema (green) and palmar psoriasis (blue). [file CUP-53-444-s003.tif]
