# Supplementary material for: Harnessing Digital Pathology Tools to Distinguish Hand Eczema From Palmar Psoriasis: A Quantitative Approach
Source: J Cutan Pathol. 2026 Feb 5;53(5):444–53. doi: 10.1111/cup.70064 (PMC13040410; doi:10.1111/cup.70064)
Supplement: Supplementary file 4 — Table S1: Subgroup analysis. Table S2: Results subgroup analysis. [file CUP-53-444-s002.docx]

**Supplementary Table 1.** Subgroup Analysis

| **Subgroup Category** | **Total Cases (n=14)** | **From Eczema (n=7)** | **From Psoriasis (n=7)** |
| --- | --- | --- | --- |
| Poor Sample Quality: Artifacts, Fragmentation, or Improper Sectioning | 6 | 3 | 3 |
| Irritated | 6 | 4 | 2 |
| Impetiginized/Eczematized | 2 | 0 | 2 |

**Supplementary Table 2:** Results Subgroup analysis

| **Parameter** | **Group** | **Mean** | **Std. Error Mean** | **ROC/AUC** | **p-Value (t-test)** | **n** |
| --- | --- | --- | --- | --- | --- | --- |
| Elongation of the rete ridges (µm) | Eczema | 311.48 | 24.86 | 0.679 | 0.009 | 35 |
|  | Psoriasis | 400.73 | 27.55 |  |  | 31 |
| Suprapapillary epidermal thickness (µm) | Eczema | 79.31 | 3.37 | 0.734 | <0.001 | 71 |
|  | Psoriasis | 56.91 | 4.20 |  |  | 54 |
| Width of the parakeratosis (µm) | Eczema | 1763.07 | 260.93 | 0.516 | 0.221 | 35 |
|  | Psoriasis | 2130.44 | 412.23 |  |  | 30 |
| Capillary diameter (µm) | Eczema | 104.84 | 9.38 | 0.720 | 0.072 | 24 |
|  | Psoriasis | 274.51 | 110.45 |  |  | 18 |
| Width of the rete ridges (µm) | Eczema | 81.77 | 3.42 | 0.692 | <0.001 | 70 |
|  | Psoriasis | 64.01 | 3.26 |  |  | 54 |
| Ratio Stratum granulosum to epidermis (µm²/µm²) | Eczema | 0.13 | 0.01 | 0.514 | 0.013 | 71 |
|  | Psoriasis | 0.10 | 0.01 |  |  | 52 |
| Ratio Stratum corneum to epidermis (µm²/µm²) | Eczema | 0.64 | 0.06 | 0.513 | 0.407 | 71 |
|  | Psoriasis | 0.68 | 0.14 |  |  | 54 |
| Ratio of spongiosis to epidermis (µm²/µm²) | Eczema | 188.18 | 3.21 | 0.579 | 0.411 | 72 |
|  | Psoriasis | 189.08 | 1.81 |  |  | 55 |
| CD3 (cells/µm²) | Eczema | 60.74 | 9.46 | 0.515 | 0.273 | 64 |
|  | Psoriasis | 53.17 | 7.52 |  |  | 52 |
| CD15 (cells/µm²) | Eczema | 38.69 | 19.46 | 0.686 | 0.168 | 55 |
|  | Psoriasis | 63.45 | 16.11 |  |  | 49 |
| CD20 (cells/µm²) | Eczema | 2.80 | 0.94 | 0.617 | 0.070 | 67 |
|  | Psoriasis | 5.82 | 2.01 |  |  | 49 |
| PHH3 (cells/µm²) | Eczema | 19.92 | 2.28 | 0.626 | 0.011 | 67 |
|  | Psoriasis | 31.04 | 4.15 |  |  | 53 |
| S100 (cells/µm²) | Eczema | 105.93 | 15.09 | 0.586 | 0.018 | 61 |
|  | Psoriasis | 67.23 | 10.12 |  |  | 49 |
